# Miscibility and Phase Separation in PMMA/SAN Blends Investigated by Nanoscale AFM-IR

**DOI:** 10.3390/polym13213809

**Published:** 2021-11-04

**Authors:** Julia Resch, Julia Dreier, Christian Bonten, Marc Kreutzbruck

**Affiliations:** Institut für Kunststofftechnik, University of Stuttgart, Pfaffenwaldring 32, 70569 Stuttgart, Germany; julia.dreier@ikt.uni-stuttgart.de (J.D.); christian.bonten@ikt.uni-stuttgart.de (C.B.); marc.kreutzbruck@ikt.uni-stuttgart.de (M.K.)

**Keywords:** PMMA/SAN blends, miscibility, phase separation, nanoscale AFM-IR

## Abstract

The miscibility and phase separation of poly(methyl methacrylate) (PMMA) and styrene-acrylonitrile (SAN) have already been investigated using various methods. However, these methods have limitations that often result in inconsistent characterization. Consequently, the reasons for the dependence of miscibility on composition as well as on processing temperature have not yet been proved. The phase separation of PMMA/SAN blends was therefore investigated for the first time using a novel technique, nanoscale AFM-IR. It couples nanoscale atomic force microscopy (AFM) with infrared (IR) spectroscopy. Therefore, the phase morphology can be chemically identified and precisely classified within the nm-regime. The PMMA/SAN blends, on the other hand, were analyzed of their changes in morphology under different thermal treatments. It was possible to visualize and define the phase separation, as well as dependence of the miscibility on the mixing ratio. In the miscible domain, no two individual phases could be detected down to the nanometer range. It was shown that with increasing temperature, the morphology changes and two different phases are formed, where the phase boundaries can be sharply defined. The onset of these changes could be identified at temperatures of about 100 °C.

## 1. Introduction

Blending of polymers allows the development of novel materials with customized property profiles. It is a cost-effective modification by which characteristics such as processability or mechanical properties of polymers can be adjusted [1]. It is assumed that the miscibility of the both components used for mixing also has an effect on the subsequent properties of the blend. Polymer blends consist of at least two different polymers. However, these are not polymerized during synthesis, e.g., by a chain growth reaction as in the case of copolymers, but two polymers are mixed together. In this way, often only physical bonds are formed.

Thermodynamically, a basic distinction can be made between miscible, immiscible, and partially miscible blends. The most frequently encountered polymer mixtures are immiscible or only partially miscible. In the partial miscibility range, the phase behavior exhibits a miscibility gap. It shows phase separation during heat treatments, which is characterized by a lower segregation temperature. If one is below this temperature, only one phase is formed, and the substances are completely miscible with each other in all concentration ratios [2].

In the physical sense, miscibility means homogeneity down to the molecular level [3]. Thus, only one phase is formed. Full miscibility in polymer blends is rare. Due to the usually very long polymer chains, a complete intermixing is difficult to achieve. In general, the formation of a miscible blend depends on the exothermic heat of mixing, which is usually determined by the intermolecular interactions such as dipolar, hydrogen bond, or charge transfer interactions [4]. On the contrary, intramolecular repulsive forces can also be the reasons for miscibility of two or more phases. They are observed for copolymers as a blend component like styrene acrylonitrile (SAN) [5,6].

A well-known example is the blend of poly(methyl methacrylate) (PMMA) and SAN. The mixing behavior of the two blend partners is primarily dependent on the acrylonitrile (AN) content of SAN. Various values for miscibility can be found in the literature. According to Kressler, blends with an AN content of 26 wt.% to 30 wt.% show a miscibility gap [7]. Stein, in turn, reports that with an AN content between 9 wt.% and 26.5 wt.% a complete miscibility and from 26.6 wt.% to 29.5 wt.% a miscibility with a miscibility gap occurs [8]. Suess and Fowler specify a miscible range from 9 wt.% to 33 wt.% [5,9].

Moreover, the reason for this occurring miscibility is debated. However, neither of the two components of the copolymer, i.e., polystyrene (PS) or polyacrylonitrile (PAN), shows a miscibility with PMMA, implying the absence of a specific interaction between the two polymer phases. Various models have been used to explain these effects through intramolecular repulsive forces within the co-polymers. Paul et al. for example suggested that miscibility arises from the repulsive forces between the acrylonitrile and styrene groups of the SAN copolymer [10,11]. Feng, on the other hand, disagreed with this theory and assumed that the miscibility in this system is due to attractive interactions between the carbonyl groups of PMMA and the phenyl groups of SAN [12].

Kumaraswamy did not find strong interactions between PMMA and SAN chains in his studies and thus could not confirm Feng’s theory [4]. Suess and Kumaraswamy also reported that the miscibility is dependent on the composition of the mixing partner and changes with the blend ratio of the blend components [4,5].

The miscibility of plastic blends was determined by many methods; for example, by cloud point measurements [9,13] or infrared microscopy [14,15]. In general, it is observed that amorphous, miscible blends exhibit transparency. Therefore, non-miscibility of the blends is easily detected visually because phase separation causes light scattering. However, simple visual inspection is inaccurate and not reliable as the domains created by phase separation may be very small compared to the wavelength of light or have similar refractive indices [10]. Other imaging techniques such as microscopy have their limitations since amorphous polymers have no molecular superstructures that could be visualized.

One of the most commonly used methods to determine miscibility is the measurement of the glass transition temperature [16,17]. The glass transition is the reversible transition of amorphous materials in which the polymer chains change from a solid and “glassy” state to a soft and viscous state, as a result of increased Brownian molecular motion. The glass transition temperature is usually determined by differential scanning calorimetry (DSC) [1,2]. For miscible blends, it is assumed that there is a composition-independent glass transition temperature. Non-miscible blends, on the other hand, show two glass transitions characteristic of each phase in the mixture. Yet, glass transition measurements are only of limited use for evaluating miscibility, since in some cases the glass transition temperatures of two immiscible blend partners are too similar that they cannot be adequately resolved [3].

There are some other efforts to characterize the phase behavior. For example, the rheological property behavior and morphology near the miscibility gap have been described [18,19,20] or phase separation has been studied using free volume theory [4].

A less indirect way of analyzing miscible blends is to analyze the morphology using optical techniques such as transmission electron microscopy (TEM) [21] or scanning electron microscopy (SEM). Due to its high surface sensitivity; however, atomic force microscopy (AFM) is the most commonly used technique [22,23]. With AFM, surface structures can be imaged at a resolution down to the nanoscale and surface forces can be measured [24]. It is thus possible to record the phase morphologies of polymer blends. A disadvantage of these methods for the investigation of miscibility and phase separation is that no exact analysis of the composition of the blend morphology, i.e., no reliable chemical determination of the individual phases, can take place [24].

In this work, a novel method is used to investigate the morphologies of PMMA/SAN blends annealed at different temperatures. For the first time, the morphologies are imaged and chemically mapped by this method. Thus, the phase separation will be precisely characterized. Based on these results, the phase separation phenomenon and the mixing principle of the PMMA/SAN system are discussed.

## 2. Materials and Methods

### 2.1. Materials and Blend Preparation

In this study, PMMA (Plexiglas 7N from Röhm, Darmstadt, Germany), and SAN (Luran from Ineos Styrolution, Ludwigshafen/Rhein, Germany) with an acrylonitrile content of 30 wt.% were used. Films with a blend composition of 0 wt.%, 30 wt.%, 50 wt.%, 70 wt.%, and 90 wt.% SAN were produced. For this purpose, the two blending partners were dissolved in tetrahydrofurane at 60 °C and poured onto a petri dish. The solvent was evaporated for 48 h at room temperature. The resulting films of the blends, which have a thickness between 200 and 300 µm, were vacuum-dried at 50 °C for 48 h. Subsequently, the films were annealed at different temperatures (ranging from 80 °C to 180 °C) for 24 h. To characterize the prepared and annealed blend films, they were prepared as thin sections with an ultramicrotome Ultracut E (Leica, Wetzlar, Germany), with an average thickness of 100 nm.

### 2.2. Nanosale AFM-IR Measurements

**Figure 1:**
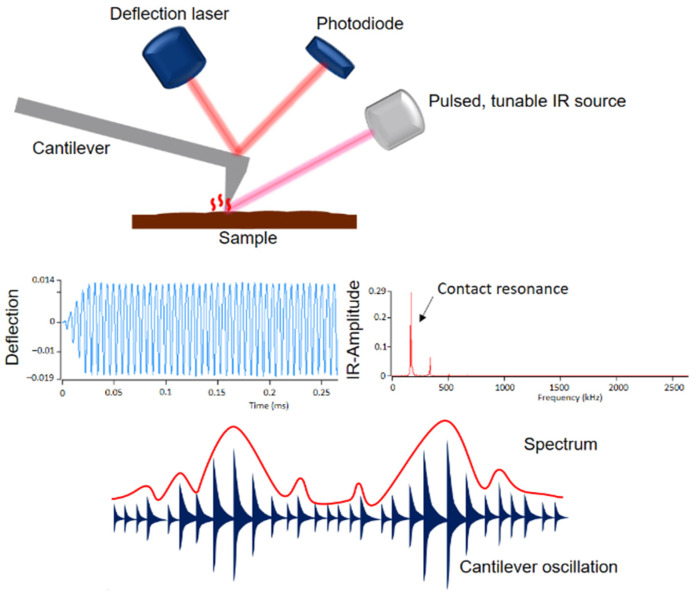
Setup and measurement principle of AFM-IR.

A novel, powerful technique is an atomic force microscope (AFM) coupled with infrared (IR) spectroscopy (Bruker, Billerica, MA, USA). AFM is an important tool in surface and polymer chemistry. It allows nanoscale measurements that provide information on topography, morphology, crystallization of polymers, friction behavior and adhesion. The measurement principle is based on scanning the sample surface with a pyramid-shaped nanoscopic tip. Even though nanometer-scale measurements are possible with AFM, it has a major weakness: information about the chemical composition of materials is not possible [25,26]. However, combining AFM with IR spectroscopy can overcome this drawback. Figure 1 shows the setup and measurement principle of the AFM-IR technique.

A pulsed tunable laser is focused on the sample near the tip of the cantilever. When the laser is tuned to an absorption band of the sample, photothermal expansion of the sample occurs at the absorbing sites. This expansion causes an oscillation of the cantilever, which is proportional to the IR absorption. The amplitude of this oscillation results in an IR spectrum as a function of the wavenumber. In addition to the IR spectra, the AFM-IR provides IR images, the so-called chemical mapping, with which the individual components can be identified [27,28,29,30,31,32]. The phase morphologies were examined with an Ansys NanoIR3 AFM-IR (Bruker, Billerica, MA, USA). A contact cantilever was used for data acquisition, which was operated in contact AFM mode. Scans were performed at wavelengths of 1730 cm^−1^, 1600 cm^−1^ and 1450 cm^−1^. Ratio images were created from the individual scans.

In this work, the individual scans are put into ratio and the height images of the individual measurements are subtracted. In addition, individual spectra were recorded locally. The images generated were subsequently analyzed using Analysis Studio software (Anasys Instruments, Santa Barbara, CA, USA) [27].

## 3. Results

All PMMA/SAN blend samples, annealed at different temperatures, were characterized. For the phase separation study, only the samples with 50 wt.% SAN annealed at different temperatures are considered. The produced films are completely transparent after vacuum drying as well as after the film was annealed at 80 °C. The transparency indicates a complete mixing of the two blending components PMMA and SAN. Visual inspection of the films stored at 100 °C and 120 °C also shows high transparency; however, a somewhat weaker opaque gloss can be seen at 100 °C and a somewhat stronger opaque sheen at 120 °C. This sheen is not visible in the films stored at room temperature. It is possible that the morphology of the blends has already started to change.

At 150 °C, the films are opaque without any transparent areas. The films reveal that the morphology has changed significantly compared to the films that were not thermally treated. At 160 °C and 180 °C, the films are completely opaque as well. The formerly transparent films have become completely opaque. A phase separation seems to have taken place. Though, phase separation and the associated clouding of the films can clearly be seen, it is not possible to determine exactly at what temperature phase separation occurred.

**Figure 2:**
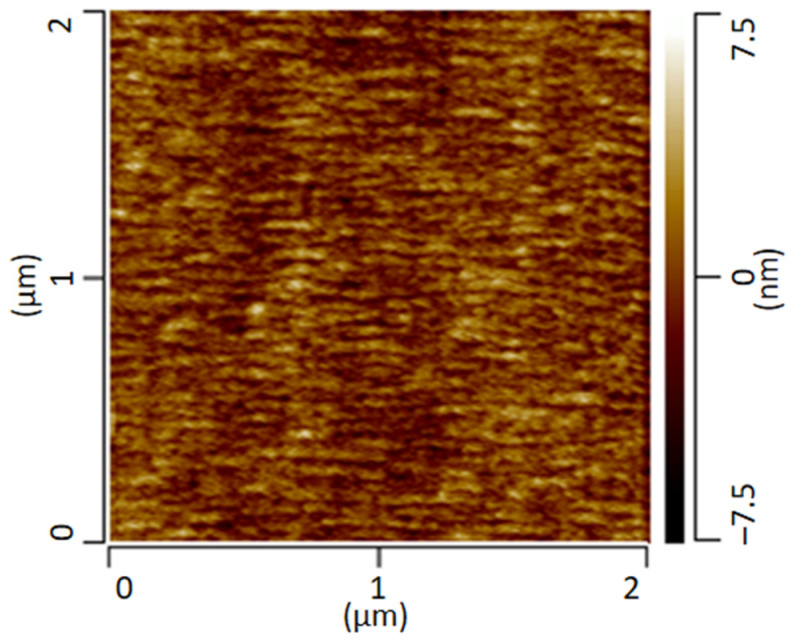
AFM height image of a PMMA/SAN blend measured in contact mode.

The following Figure 2 shows the AFM height-image of the transparent film of the PMMA/SAN blend stored at room temperature. The AFM image provides information about the height topology and morphology. The image shows a conspicuous morphology with small domains of a few nanometers. However, a clear assignment of the individual phases is not possible.

**Figure 3:**
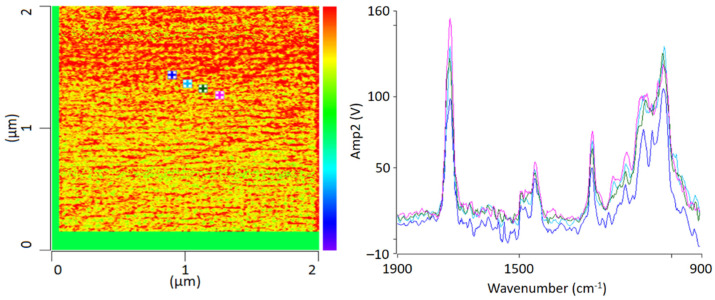
IR-AFM ratio mapping image of 1730 cm^−1^ and 1600 cm^−1^ of a PMMA/SAN film; samples stored at room temperature (**left**); AFM-IR spectra (**right**) taken spectra taken at the positions indicated (+) in the ratio image on the left.

For chemical identification by IR-AFM, the sample was scanned at the wavenumbers of the characteristic bands of PMMA (1730 cm^−1^, 1450 cm^−1^) and SAN (1600 cm ^−1^ and 1490 cm^−1^) using the IR laser. Afterwards, the images of 1730 cm^−1^ and 1600 cm^−1^ were related to another. Height topology was subtracted in order to eliminate possible influences. For comparison, Figure 3 shows an IR-AFM ratio image of the same section as Figure 2.

As shown, the images of the wavenumbers 1730 cm^−1^ and 1600 cm^−1^ have been put into ratio (wavenumber characteristic for PMMA and SAN, respectively). The yellow-colored areas in the image thus mean that there is more PMMA than SAN at these locations respectively, i.e., the probability for the wavenumber 1730 cm^−1^ is greater. The red parts can thus be assigned to a dominance of SAN. The information here is given in terms of probabilities, since the oscillation caused by the excitation of the sample does not only affect the location of the cantilever, but also everything in the immediate surroundings. The thickness of the thin section also plays a major role here, since excitation can also occur in the vertical direction.

AFM-IR allows the chemical identification of the imaged morphologies in a very high resolution for the first time. It is evident that the individual phases cannot be clearly separated from each other. A random morphology and very small domains can be seen which are in a small nanometer range. In addition to the ratio image, individual spectra were recorded at various points (Figure 3 left). The spectra show that the individual polymers cannot be differentiated; at any point the measurements were taken, the spectra look similar and show the characteristic bands of PMMA as well as of SAN. The two phases are mixed with each other at a level down to a few nanometers’ resolution.

**Figure 4:**
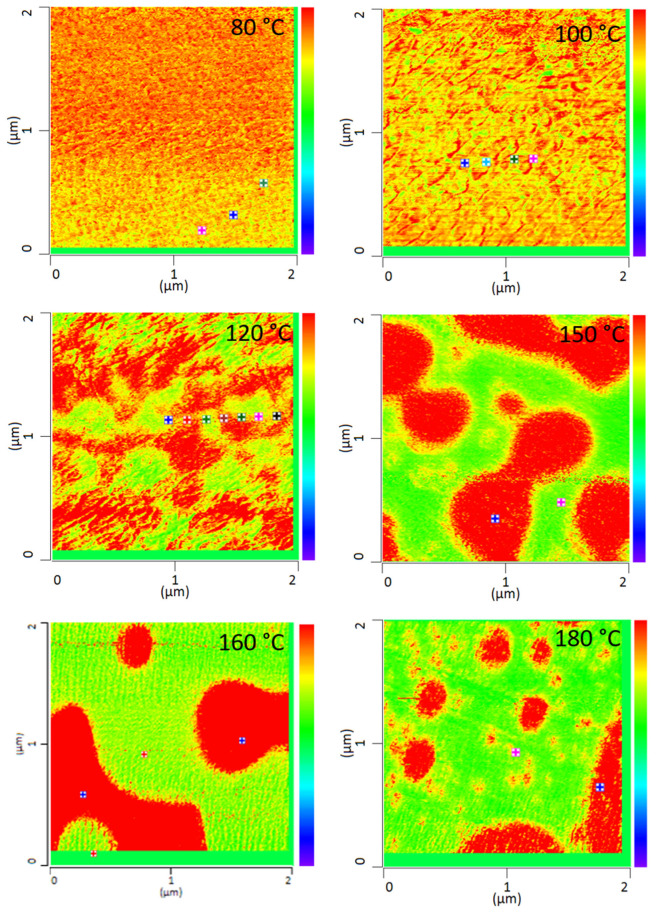
IR-AFM ratio mapping image of 1730 cm^−1^ and 1600 cm^–1^ of PMMA/SAN 50/50; blend samples annealed at different temperatures. The positions indicated (+) show where the spectra were taken in Figure 5.

All PMMA/SAN blend samples annealed at different temperatures were characterized by IR-AFM and are shown in Figure 4. Scans were made at 1730 cm^−1^ and 1600 cm^−1^ and the ratio images were calculated respectively.

**Figure 5:**
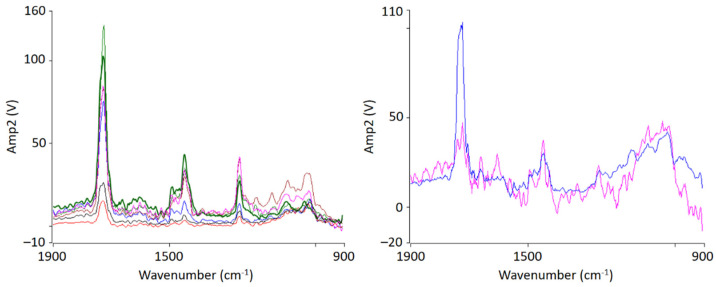
Localized nanoscale AFM-IR spectra of PMMA/SAN 50/50 blend samples at 120 °C (**left**) and 150 °C (**right**) from the indicated position shown in Figure 4. The colors of the spectra lines correspond to the color of the markings.

The film annealed at 80 °C shows a morphology similar to the film stored at room temperature. Very small randomly arranged domains are visible which are in the small nanometer range. The individual domains cannot be clearly identified. A complete mixing of PMMA and SAN is present. At 100 °C, in comparison, a clear enlargement of the domains can be seen. Small domains can be identified, most of which can be assigned to SAN. The individual phases are therefore already beginning to separate. Nevertheless, the measured spectra cannot yet be clearly assigned to the individual components, but contain the peaks of both SAN and PMMA. At 120 °C continuous phase regions are formed. However, the interfaces of these domains are rather indistinct and diffuse. Although the film is still optically transparent, a clear change in morphology has taken place. However, the fact that complete phase separation has not yet occurred can also be seen in the measured spectra in Figure 5. No clear assignment to a phase can be seen. The spectra show the characteristic bands for both PMMA and SAN.

At 150 °C, a clear co-continuous structure of the morphology can be seen. The phase separation has progressed. A clear, sharp boundary between the two phases can be seen, the domains are between several hundred nanometers and a few micrometers wide. For the first time, a clear identification of the individual phases via their spectra is possible. As can be seen in Figure 5 on the right hand, the spectrum of the red domains clearly represents the spectrum of PMMA with a very strong band at 1730 cm^−1^. The red domains can therefore be identified as PMMA. The spectrum of the green domains shows the spectrum of SAN with a characteristic band at 1600 cm^−1^. There is a small band at 1730 cm^−1^. This is due to the very strong intensity of the carbonyl bonds in PMMA, which therefore can also be seen in the spectrum of SAN.

With increasing temperature, phase separation progresses and morphology changes from a co-continuous structure to a dispersed morphology at 180 °C. Complete phase separation is evident here. The PMMA is dispersed in the SAN. Larger domains have formed, some of which are several micrometers in size and can be clearly separated from one another.

**Figure 6:**
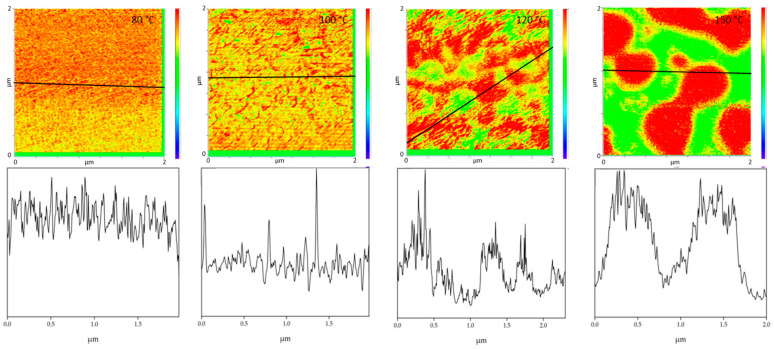
The figures show cross sections (**bottom**) taken along the black solid lines in the IR-AFM ratio images (**top**).

Comparing the phase separation results from AFM-IR with the literature, the phase separations in the literature are often determined at higher temperatures. Wen et al. investigated the phase separation of PMMA/SAN blends with an AN-content of 30 wt.% by AFM. He was able to detect a change in morphology above 150 °C [24]. AFM-in situ studies by Liao et al. [33] and You et al. [34] also found a critical surface phase separation temperature at 165 °C. In this work it was found that the morphology of the blends changes already at 100 °C and thus the phase separation starts much earlier. However, it should be noted that Wen et al. as well as Liao et al. and You et al. are dealing with thin film samples which are only 2D in nature. A film thickness of about 100 nm will inevitably lead to a classical two-dimensional sample which in turn will show significant deviations to the diffusion and phase transition kinetics of their 3D counterparts. In addition, the corresponding transport mechanisms and temperature based percolation phenomena will be highly dependent on the dimensions of the sample under test [35,36,37]. Additionally, especially for very thin films, surface adhesion and, for example, the sample substrate can also have an influence on the measurements. It was also shown that the phases can be clearly separated from each other during phase separation and the phase boundaries can be seen very sharply. Wen et al. had concluded in their investigations, that there are no clear phase boundaries at the surface. This could not be confirmed by these investigations. Figure 6 uses the cross-section to illustrate once again how clear the individual phases can be distinguished from one another at the different annealing temperatures. This also allows to derive the diameter size of the individual domains. The half-width of the phase domains is about 35 nm at 100 °C. At 120 °C the half-width of the domains is more than 5 times higher about 189 nm and at 150 °C the separate domains have a half-width of 517 nm.

**Figure 7:**
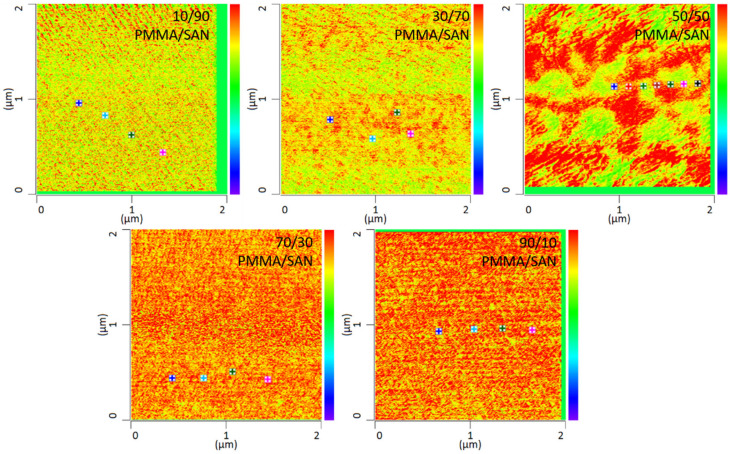
IR-AFM ratio mapping image of 1730 cm^−1^ and 1600 cm^−1^ of PMMA/SAN blend samples at different ratios annealed at 120 °C, The positions indicated (+) show where the spectra were taken.

In addition to the temperature dependency of the blends, the miscibility as a function of the mixing ratio was also investigated in this work. By way of example, various mixing ratios of PMMA and SAN at 120 °C storage are shown in Figure 7. It can be seen that the mixing ratio also has a clear influence on the mixing behavior. The blends with a mixing ratio of 10 wt.% PMMA and 90 wt.% SAN show complete miscibility of the two blend partners. At a somewhat lower SAN content of 70 wt.%, slightly larger domains are formed. At a mixing ratio of 50 wt.% PMMA and 50 wt.% SAN, a clear change in the morphology can be seen. Here, a separation of the two phases can be noted, though the phase boundaries are not sharply defined. At a higher PMMA content, separation of the phases is no longer visible. In fact, the blend is once again a complete mixture of the two phases. Complete mixing is present again. It can therefore be said that phase separation occurs at lowest temperatures with a SAN content of 50 wt.%. Thus, higher or lower SAN contents need higher temperatures to separate phases.

## 4. Conclusions

Using a novel method, the phase separation behavior of PMMA/SAN blends under thermal treatment and different mixing ratios was investigated for the first time. For the first time, it was also possible to chemically resolve the morphology and thus identify the individual phases.

The investigations have shown that nanoscale AFM-IR is very well suited for the analysis of miscibility. Nanoscale IR spectroscopy allows identification of the phase separation mechanisms on the basis of the individual phases. It was shown that in the case of complete miscibility, the individual components of the blend cannot be separated from each other and the molecules are mixed on a level of a few nanometers. In the case of phase separation, the two phases can clearly be distinguished and identified chemically. The phase boundaries are clearly recognizable and sharply separated from each other. With increasing temperature, the morphology changes from a co-continuous structure to a dispersed structure. Additionally, the onset of phase separation could be defined by a change in morphology at a temperature of 100 °C. It could also be shown that the mixing ratio has an influence on the miscibility and phase separation. If the SAN content is very high or very low, phase separation only starts at higher temperatures.

In future work, we would like to investigate other miscible and partially miscible blend systems and their phase separation. The dependence of the miscibility on the AN-content was also observed with other blend partners apart from PMMA and could thus provide further indications of the mechanisms of action. In addition, we would like to make further measurements with PMMA/SAN blends in which the composition, i.e., the proportion of PMMA-rich or SAN-rich phases, will also be measured in order to be able to draw further conclusions concerning the phase diagram. The dynamics of the phase separation will also be analyzed quantitatively in further investigations.

## Data Availability

Not applicable.
